# Changes of gut microbiota in colorectal cancer patients with *Pentatrichomonas hominis* infection

**DOI:** 10.3389/fcimb.2022.961974

**Published:** 2022-08-31

**Authors:** Hongbo Zhang, Yanhui Yu, Jianhua Li, Pengtao Gong, Xiaocen Wang, Xin Li, Yidan Cheng, Xiuyan Yu, Nan Zhang, Xichen Zhang

**Affiliations:** ^1^ Key Laboratory of Zoonosis Research by Ministry of Education, College of Veterinary Medicine, Jilin University, Changchun, China; ^2^ Second Affiliated Hospital, Jilin University, Changchun, China; ^3^ Clinical Laboratory, Jilin Cancer Hospital, Changchun, China; ^4^ Key Laboratory of Zoonosis Research by Ministry of Education, Institute of Zoonosis, Jilin University, Changchun, China

**Keywords:** colon cancer, *Pentatrichomonas hominis*, gut microbiota, 16S rRNA, high-throughput sequencing

## Abstract

*Pentatrichomonas hominis* is a parasitic trichomonads protozoa that parasitizes in the colon and cecum of humans and other animals. Our previous studies have demonstrated that infection with *P. hominis* is associated with the incidence of colon cancer (37.93%). However, the mechanism by which *P. hominis* infections increase the incidence of colon cancer remains unclear. Previous studies have suggested that certain parasites promote colon cancer by regulating gut microbiota. This study aimed to elucidate whether the association between *P. hominis* infections and the increased incidence of colon cancer is related to changes in gut microbiota. Therefore, the gut microbiota patients with colon cancer who were infected with *P. hominis* and uninfected patients with colon cancer were analyzed by 16S rRNA high-throughput sequencing. The results demonstrated that patients with colon cancer who were not infected with *P. hominis* showed increased gut bacterial diversity, a higher relative abundance of *Alcaligenes* sp., *Leucobacter* sp., *Paraprevotella* sp., *Ruminococcaceae UCG-002*, and a significant reduction in the abundance of *Veillonella* sp., compared to individuals without colon cancer. Additionally, the relative abundance of the *Ruminococcaceae UCG-002* and the *Eubacterium eligens* groups was reduced, while the relative abundance of bacteria associated with colon cancer, including *Flavonifractor* sp., *Lachnoclostridium* sp., and the *Ruminococcus gnavus* group, increased significantly in patients with colon cancer who were infected with *P. hominis*, compared to those of uninfected patients with colon cancer. In conclusion, these results suggested that *P. hominis* infections may aggravate the development of colon cancer and the findings provide new insights for subsequent in-depth studies on the pathogenesis, diagnosis, and prevention of colon cancer.

## Introduction

Colon cancer is one of the three leading causes of cancer-related deaths worldwide (Khan et al., 2022). According to recent statistics, there are approximately 376,300 new cases of colon cancer annually (Chen et al., 2016). The onset of colon cancer is influenced by several factors, including alterations in host genes, epigenetics, and environmental factors (Okugawa et al., 2015). Recent studies have demonstrated that parasitic infections could be an important factor in inducing the development of colon cancer. Studies by Choi and Sriamporn reported that *Opisthorchis viverrini* and *Clonorchis sinensis* are two biological carcinogens that can cause cholangiocarcinoma (Sriamporn et al., 2004; Choi et al., 2006). Muscheck et al. (2000) observed that chronic infection with *Schistosoma* sp. is closely related to the occurrence of bladder cancer and increased chromosomal aberrations (Muscheck et al., 2000). Studies have also reported that schistosomiasis and amebiasis increase the incidence of colorectal cancer (CRC) (Waku et al., 2005; Madbouly et al., 2007). Sulzyc-Bielicka et al. observed that infection with *Blastocystis* sp. significantly increases the incidence of colon cancer (Sulzyc-Bielicka et al., 2021). Additionally, studies on murine models of CRC have reported that infections with different intestinal parasites have variable effects on tumor initiation and formation in CRC (Hayes et al., 2017; Biton et al., 2018).

The trichomonas protozoan, *Pentatrichomonas hominis*, primarily parasitizes in the cecum or colon of humans and other mammals, including domestic animals, laboratory animals, companion animals, and wild animals and is usually transmitted through the fecal-oral route (Li et al., 2018; Li et al., 2020). The pathogenicity of *P. hominis* has been controversial for a long time. In the past, *P. hominis* was considered to be a non-pathogenic protozoa or conditional pathogen in the gut of animals. However, recent studies have detected *P. hominis* in the stool of immunocompromised animals and patients (Gookin et al., 2005; Meloni et al., 2011; Compaore et al., 2013), and its pathogenicity has gradually attracted increasing attention. A recent study by Aleksey et al. demonstrated that *Tritrichomonas musculis*, which belongs to the Trichomonas family and parasitizes in the same site as *P. hominis*, promotes the occurrence of colitis and colon cancer (Chudnovskiy et al., 2016). Our recent investigation also demonstrated that *P. hominis* infections significantly increase the incidence of colon cancer in infected individuals compared to uninfected patients (Zhang et al., 2019a). However, the mechanism underlying the increased incidence of colon cancer following *P. hominis* infections remains unclear.

Recent studies have demonstrated that gut microbiota plays an important role in the occurrence of colon cancer (Fan et al., 2021). A recent study revealed that *Fusobacterium nucleatum*, *Enterotoxigenix Bacteroides fragilis* (ETBF), *Escherichia coli*, *Salmonella* sp., *Desulfpvibrio desulfuricans*, *Enterococcus faecalis*, and their metabolites are closely related to the occurrence of colon cancer (Fan et al., 2021). Other studies have also indicated that tryptophan metabolism disorder, which is mediated by gut microbiota, plays a role in the incidence of CRC (Sun et al., 2020). With the development of bacterial detection technologies, fecal flora can be used as diagnostic markers for the non-invasive screening of CRC and colon cancer or post-chemotherapy recovery (Jang et al., 2020; Liang et al., 2020). Additionally, probiotics such as *Clostridium butyricun* inhibits intestinal inflammatory damage, increases the apoptosis of cancer cells, and reduces the incidence of intestinal cancer and tumor size by regulating gut microbiota (Liu et al., 2020). Recent studies have demonstrated that the abundance of *Fu*. sp., which is associated with the occurrence of CRC, is significantly higher in the fecal matter of newborn calves infected with *Cryptosporidium parvum* (Bashir et al., 2015; Ichikawa-Seki et al., 2019). We therefore speculated that one of the underlying reasons for the positive correlation between *P. hominis* infection and the prevalence of colon cancer is associated with an increased abundance of pathogenic bacteria associated with intestinal cancer in the gut. Therefore, the characteristics of the gut microbiota of uninfected patients with colon cancer and patients with colon cancer who were infected with *P. hominis* were investigated in this study, and the results paved the way for a new avenue of research on the pathogenesis and prevention of colon cancer.

## Materials and methods

### Fecal sample collection

Fecal samples were collected using the method described in our previous study (Zhang et al., 2019a). Briefly, 8 fecal samples were collected from patients with CRC, aged 50 to 70 years, who were infected with *P. hominis* and 8 fecal samples were collected from uninfected CRC patients from the Jilin Cancer Hospital in Changchun, China, from January to July of 2018. The control group was comprised of 9 fecal samples collected from the Second Hospital of Jilin University in Changchun, China, from local residents who had no gastrointestinal complaints or *P. hominis* infections. Fresh fecal samples were collected in individual containers. One part of the samples was frozen in liquid nitrogen and kept in -80°C for 16S rRNA gene amplicon pyrosequencing. The other portion of the samples was stored at -20°C until DNA extraction, which was generally performed within 24 h.

### Identification of *P. hominis*

*P. hominis* was identified by PCR analysis (**Supplemental Material**; **Figures S1, S2**). Also, 25 fecal samples were examined by nested PCR for detection of *Giardia duodenum* and *Cryptosporidium parvum* (**Supplemental Material**; **Figures S3–S6**). DNA was extracted from each of the fecal samples using a Fecal DNA Rapid Extraction Kit (TIANGEN, Beijing, China), according to the manufacturer’s instructions (Zhang et al., 2019a).

### MetaVx™libary construction and Illumina MiSequ sequencing

Construction of the high-throughput sequencing library and sequencing on an Illumina MiSeq platform were performed by GeneWiz (Suzhou, China). The concentration of the DNA samples was determined using a Qubit 2.0 Fluorometer (Invitrogen, Carlsbad, CA, USA). Sequencing libraries were constructed using a MetaVxTMlibrary Construction Kit (GeneWiz Inc., South Plainfield, NJ, USA). The PCR primers, 5′-CCTACGGRRBGCASCAGKVRVGAAT-3′, and 3′-GGACTACNVGGGTWTCTAATCC-5′ were used for amplifying the two highly variable regions, V3 and V4, on prokaryotic 16S rDNA. In addition, an INDEX connector was added to the end of the 16S rDNA PCR product for next-generation sequencing (NGS). The quality and concentration of the library were detected using an Agilent 2100 bioanalyzer (Agilent Technologies, Palo Alto, CA, USA) and a Qubit 2.0 fluorometer (Invitrogen, Carlsbad, CA), respectively. After the DNA library was mixed, PE250/300 double-ended sequencing was performed according to the instructions of Illumina Miseq (Illumina, San Diego, CA, USA). The sequence information was read using the Miseq Control Software (MCS).

### Analysis of sequencing data

The reads obtained by double-ended sequencing were first assembled and linked by pair assembly. The chimeric sequences were removed after mass filtration and the sequences obtained were used for analyzing the operational taxonomic units (OTUs). Sequence clustering was performed with VSearch version 1.9.6 (sequence similarity of 97%), and the SILVA 132 16S rRNA reference database was used for comparison (Quast et al., 2013; Yilmaz et al., 2014; Rognes et al., 2016). The Ribosomal Database Program (RDP) classifier Bayesian algorithm was used for taxonomic analysis of the representative OTU sequences and the community composition of each sample was determined under different species classification levels (Wang et al., 2007; Wang et al., 2012). Based on the results of OTU analysis, the random sampling method was adopted for calculating the α diversity from the Shannon and Chao1 indices. The unweighted UniFrac method was used for analyzing the significant differences in the microbial communities among the samples. A principal coordinate analysis (PCoA) map, based on a Bray-Curtis distance matrix representing the differences in gut microbiota composition between samples, was generated and visualized for estimating the β diversity.

### Statistical analyses

All the statistical analyses were performed in SPSS Statistics software, version 20.0 (IBM, Armonk, NY, USA). Statistical significance was estimated using Pearson’s Chi-square test or Fisher’s exact test. All statistical tests were two-sided. *P* < 0.05 was considered to be statistically significant.

## Results

### 16S rDNA amplicon sequencing of microbiota in fecal samples

**Figure 1:**
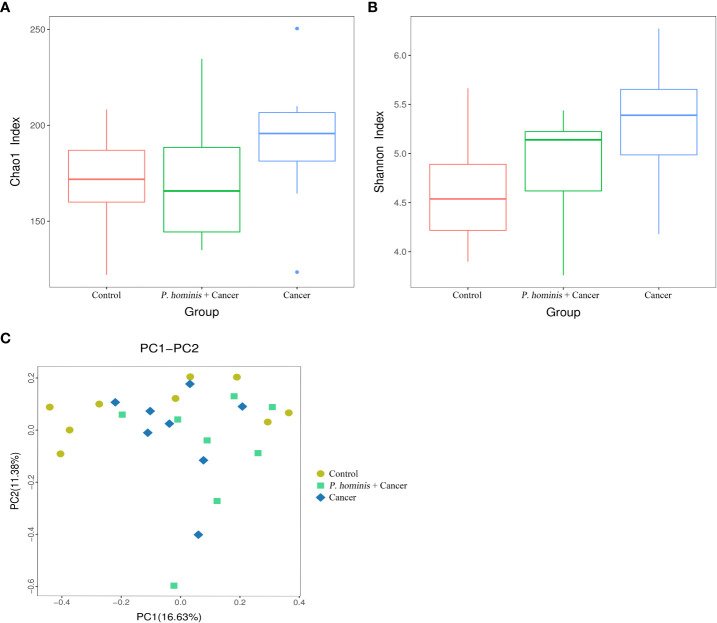
Structure, richness, and diversity of the gut bacterial community of the control group (Control), uninfected patients with colon cancer (Cancer), and patients with colon cancer who were infected with *P. hominis* (*P. hominis+*cancer). **(A)** Comparison of the richness of the microbiota in the fecal samples among the Control, Cancer, *P. hominis+*cancer groups based on the Chao1 index. **(B)** Comparison of the fecal diversity of the microbiota among the Control, Cancer, and *P. hominis+*cancer groups based on the Shannon index. **(C)** PCoA of gut microbiota among the Control, Cancer, and *P. hominis+*cancer groups. *P* < 0.05 indicates that the difference between two groups was statistically significant.

By nested PCR, 25 fecal samples (including 9 control samples, 8 samples from patients with colon cancer who were infected with *P. hominis*, and 8 samples from uninfected patients with colon cancer) were identified and no *G. duodenum* or *C. parvum* infections were detected (**Supplemental Material**; **Figures S3–S6**). All samples were subjected to 16S rRNA high-throughput sequencing analysis. A total of 3,081,004 reads were obtained, with an average of 123,240 reads per sample. OTU clustering of the non-repetitive sequences was performed based on 97% similarity. The representative OTU sequences, with the exception of chimeras, were obtained during clustering and all the optimized sequences were mapped to the representative OTU sequences to obtain a total of 351 sequences. The richness and diversity of the gut microbiota were analyzed based on the Chao1 and Shannon indices, respectively. The results demonstrate that the richness and diversity of gut microbiota increased in patients with colon cancer compared to those of healthy individuals. The value of the Shannon index suggested that the diversity of the gut microbiota of patients with colon cancer was significantly higher than that of the control group. In addition, analysis of the Chao1 index revealed that alterations in the richness of the gut microbiota of patients with colon cancer were higher than those of healthy individuals (**Figures 1A, B**). However, the richness of the gut microbiota was reduced while the diversity of the microbiota was not significantly altered in patients with colon cancer who were infected with *P. hominis*, compared to patients uninfected with colon cancer (**Figures 1A, B**). Additionally, the results of PCoA analysis revealed that there were no significant differences in the structure of the gut microbiota among the control group, the colon cancer group infected with *P. hominis*, and the uninfected colon cancer group (**Figure 1C**).

### Comparison of gut microbiota at phylum and genus levels

**Figure 2:**
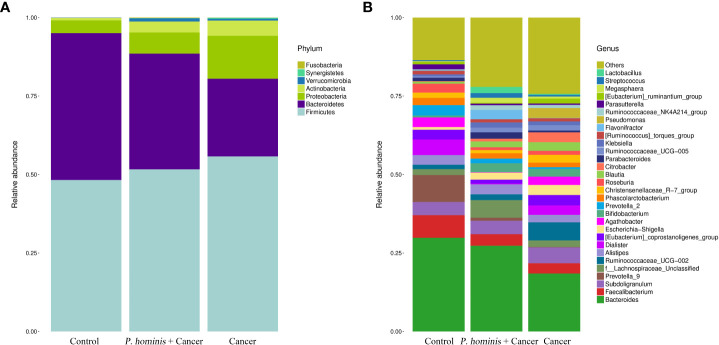
Comparison of gut microbiota at phylum and genus levels. **(A)** Relative abundance of the top 7 phyla in the fecal samples of the Control, Cancer, and *P. hominis+*cancer groups. **(B)** Relative abundance of the top 30 genera in the fecal samples of the Control, Cancer, and *P. hominis+*cancer groups.

At the phylum level, the gut microbiota of the three different groups was dominated by Firmicutes, Bacteroidetes, Proteobacteria, and Actinobacteria (**Figure 2A**). At the genus level, the bacteria mainly included *Bacteroides* sp., *Faecalibacterium* sp., *Subdoligranulum* sp., *Prevotella 9*, *f Lachnosporaceae* unclassified, *Ruminococcaceae UCG-002*, *Alistipes* sp., *Dialister* sp., the *Eubacterium coprostanplgenes* group, *Es*. sp., and *Shigella* sp. (**Figure 2B**).

### Changes in gut bacterial community among different groups

**Figure 3:**
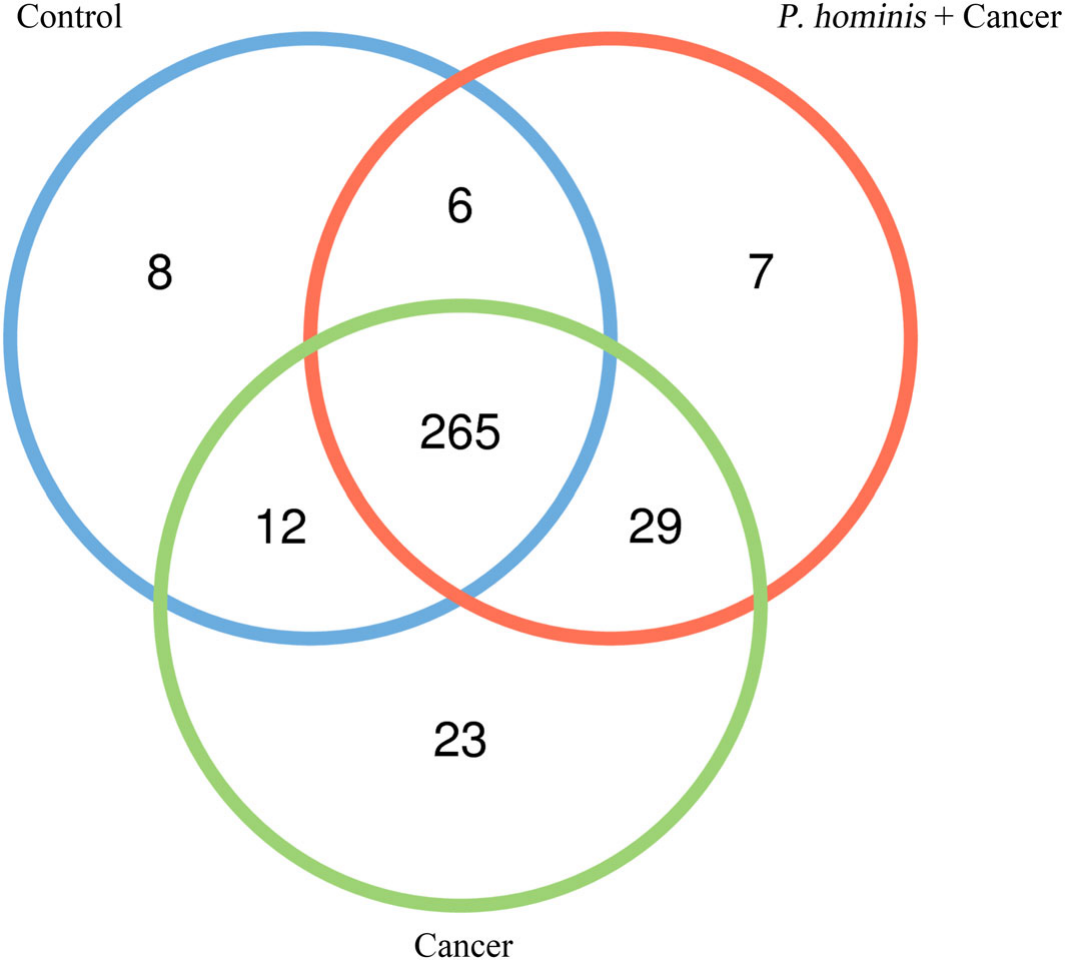
Venn diagram based on the OTUs in the Control, Cancer, and *P. hominis+*cancer groups.

In order to analyze whether a unique bacterium is associated with the occurrence of colon cancer, we analyzed the core genera that were present in the guts of the control group, the colon cancer group infected with *P. hominis*, and the uninfected colon cancer group, using Venn diagrams. The three groups shared a total of 265 core OTUs. The core genera accounted for 91.0%, 80.55%, and 86.31% of the gut bacteria in the control group, the uninfected colon cancer group, and the colon cancer group infected with *P. hominis*, respectively (**Figure 3**). These results indicated that infection with *P. hominis* may affect the occurrence of colon cancer by affecting the abundance of gut microbiota.

**Figure 4:**
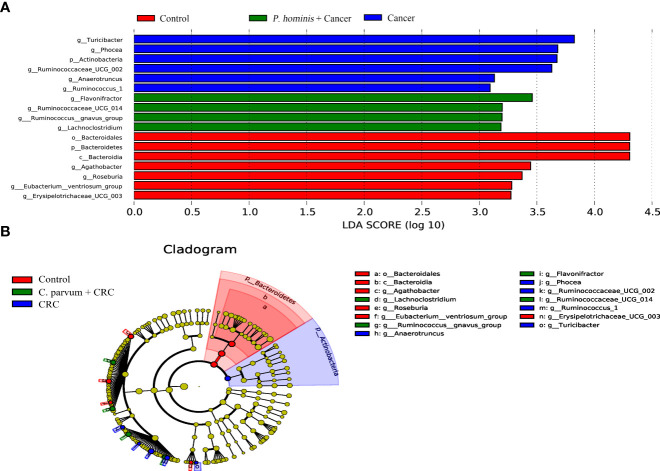
The LEfSe algorithm was used to analyze the OTU tables for identifying the taxa that best represented each biological class. **(A)** Cladogram depicting the phylogenetic distribution of the bacterial lineages in the Control, Cancer, and *P. hominis+*cancer groups. **(B)** Circles from the inside out indicate phylogenetic levels from genus to phylum.

In order to further identify the differential bacteria in the guts of uninfected patients with colon cancer and patients with colon cancer who were infected with *P. hominis*, the biomarkers in the gut bacteria were analyzed among the control group, the colon cancer group infected with *P. hominis*, and the uninfected colon cancer group, using the LDA effect size (LEFse) algorithm. As depicted in **Figure 4**, the abundance of *Actinobacteria* sp., *Turicibacter* sp., *Phocea* sp., *Ruminococcaceae UCG_002*, *Anaerotruncus* sp., and *Ruminococcus 1* was higher in the guts of patients with colon cancer who were not infected with *P. hominis*, compared to that of the control group.

**Figure 5:**
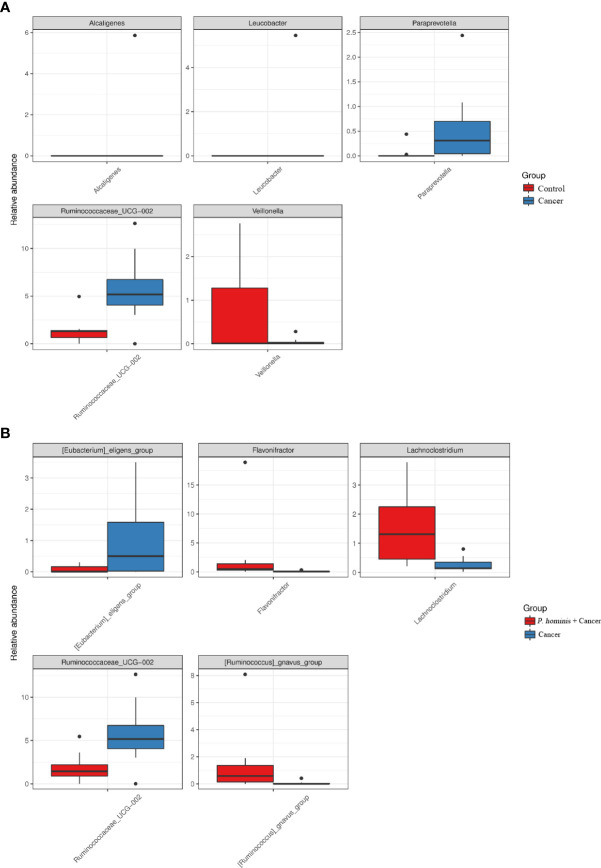
Comparison of the relative abundance of bacterial genera among the Control, Cancer, and *P. hominis+*cancer groups. **(A)** Comparison of the relative abundance of bacterial genera between the Control and Cancer groups. **(B)** Comparison of the relative abundance of bacterial genera between the Cancer and *P. hominis+*cancer groups.

Additionally, the relative abundance of *Flavonifractor* sp., *Ruminococcaceae UCG_014*, the *R*. *gnavus* group, and *Lachnoclostridium* sp. was higher in patients with colon cancer who were infected with *P. hominis*, compared to that of patients with colon cancer who were not infected with *P. hominis*. Additionally, the results of Metastats analysis revealed that the relative abundance of *Alcaligenes* sp., *Leucobacter* sp., *Paraprevotella* sp., and *Ruminococcaceae UCG-002* was significantly increased, and the relative abundance of *Veillonella* sp. was significantly reduced in the control group, compared to that of uninfected patients with colon cancer (**Figure 5A**). The relative abundance of *Eu*. *eligens* and *Ruminococcaceae UCG-002* was significantly lower, while the relative abundance of *Fl*. sp., *La*. sp., and the *R. gnavus* group was significantly higher in the guts of patients with colon cancer who were infected with *P. hominis*, compared to that of uninfected patients with colon cancer (**Figure 5B**).

## Discussion

Colon cancer is a type of adenocarcinoma that can develop into a malignant tumor if treated improperly. Colon cancer is the third leading cause of cancer-related deaths worldwide (Khan et al., 2022). The occurrence of colon cancer is related to numerous factors, including genetic factors, environmental factors, infections with bacteria such as *Helicobacter pylori*, and changes in gut microbiota. Recent studies have reported that parasitic infections are an important factor in the induction of colon cancer. Studies have confirmed that *O*. *viverrini* and *C*. *sinensis* are associated with the development of cholangiocarcinoma (Sriamporn et al., 2004; Choi et al., 2006), and chronic *Sc*. sp. infections may be an important cause of bladder cancer (Muscheck et al., 2000). Numerous studies have also demonstrated that schistosomiasis, amebiasis, and infections with *Bl*. sp. are closely related to the occurrence of CRC (Waku et al., 2005; Madbouly et al., 2007; Sulzyc-Bielicka et al., 2021). *P. hominis* is a protozoa that usually parasitizes in the cecum or colon of humans and other mammals (Li et al., 2020). *P. hominis* has been generally considered to be a non-pathogenic commensal parasite or conditional pathogen of animal intestines (Choi et al., 2006). However, recent reports have demonstrated that *P. hominis* is usually detected in the guts of individuals with weak immunity (Gookin et al., 2005; Meloni et al., 2011; Compaore et al., 2013). A previous study also reported that *Tr*. *musculis*, which belongs to the Trichomonas family and parasitizes in the same site as *P. hominis*, can promote the development of colon cancer (Chudnovskiy et al., 2016). Our recent study also revealed that infection with *P. hominis* is closely related to the incidence of colon cancer (Zhang et al., 2019a); however, the underlying mechanism remains to be elucidated.

Recent studies have demonstrated that certain parasites can affect the occurrence of colon cancer by regulating gut microbiota (Dheilly et al., 2019). It has been reported that infection with *C*. *parvum* induces disorders of gut microbiota and promotes pathological damage to the intestinal epithelium (Rahman et al., 2020). Studies have also reported that the abundance of *Fu*. sp. is significantly higher in the fecal microbiota of neonatal calves infected with *C. parvum* (Ichikawa-Seki et al., 2019), and *Fu.* sp. plays an important role in the occurrence of CRC (Bashir et al., 2015). By establishing a murine model of *Giardia* sp. infection, Barash et al. (2017) observed that the diarrhea induced by *G*. sp. may be associated with the colonization and proliferation of *G*. sp. in the small intestine, which disrupts the ecological balance of symbiotic intestinal microorganisms (Bashir et al., 2015). Furthermore, Methot and Alizon (2014) proposed that the interactions among the pathogen, host, and gut microorganisms need to be considered when studying the pathogenicity or virulence of certain pathogens (Methot and Alizon, 2014). We therefore speculated that the association between *P. hominis* infections and the high incidence of colon cancer could be associated with changes in gut microbiota. Therefore, the intestinal flora of patients with colon cancer who were infected with *P. hominis*, and patients with colon cancer who were not infected with *P. hominis*, were analyzed in the present study. The results demonstrated that the structure of the gut microbiota of patients with colon cancer was significantly different from that of healthy individuals. Uninfected patients with colon cancer had a higher relative abundance of *A*. sp., *Le*. sp., *Pa*. sp., and *Ruminococcaceae UCG-002*, compared to that of individuals without colon cancer. In addition, the relative abundance of *Fl*. sp., *La*. sp., and the *R*. *gnavus* group, which are associated with the development of colon cancer, was significantly increased in patients with colon cancer who were infected with *P. hominis*, compared to that of uninfected patients with colon cancer. These results indicated that infection with *P. hominis* increases the risk of prevalence of colon cancer, which could be attributed to the increased abundance of pathogenic intestinal bacteria associated with the development of colon cancer.

The gut is the largest immune organ in the body. Gut microbiota comprise a large number of microbial communities that colonize the intestine, and is of great important to the health of human beings. With the technological innovations in microbiota detection, the importance of gut microbiota in colon cancer has been frequently confirmed. Numerous studies have reported that the gut microbiota of patients with CRC undergoes significant alterations and disorders in gut microbiota aggravate the development of CRC (Chattopadhyay et al., 2021). We observed that the relative abundance of *A*. sp., *Le*. sp., *Pa*. sp., and *Ruminococcaceae UCG-002* in the intestine of patients with colon cancer who were not infected with *P. hominis* was significantly higher than that of individuals without colon cancer. *A*. sp. is an opportunistic pathogen that exists in gut-associated lymphoid tissues, including Peyer’s patches (Shibata et al., 2018). A study of 124 samples of mucosa-associated lymphoid tissue (MALT) of individuals from six European countries revealed that *A*. *xylosoxidans* is associated with MALT (Adam et al., 2014). Previous studies have also demonstrated that *A*. sp. is associated with the occurrence of pancreatic cancer and hematological malignancies (Aisenberg et al., 2004; Yilmaz et al., 2006). *Le*. sp. is a gram-positive aerobic bacillus without spores and exists in soils, animal intestines, wastewater, and fermented seafoods (Hyun et al., 2021). Studies have demonstrated that *Le*. sp. can invade the cecum and induce the death of parasitic worms (Hodgkin et al., 2013). *Pa*. sp. is a short-chain fatty acid (SCFA)-producing bacterium that thrives in the intestine and it has been reported that *Pa*. sp. is associated with the repair of the intestinal mucosa (Hattori et al., 2020). However, another study revealed that an increase in the abundance of *Pa*. sp. in the intestine may promote the occurrence of CRC (Luo et al., 2020). *Ruminococcaceae* is associated with the maintenance of intestinal health and the quantity of active enzymes for carbohydrate metabolism in the intestine (Zhang et al., 2019b). The abundance of *R*. sp. in the guts of patients with stage I CRC has been observed to be significantly higher than that of healthy individuals (Sheng et al., 2019). A previous study revealed that the increased abundance of Ruminococcaceae bacteria in the intestine of overweight and obese women in early pregnancy is attributed to the poor metabolic health of these individuals (Gomez-Arango et al., 2016). *Ruminococcaceae UCG-002* and *Ruminococcaceae UCG-014* are known to produce butyric acid (Huws et al., 2011). The abundance of *Ruminococcaceae UCG-014* increases significantly in the intestine of mice with dextran sodium sulfate (DSS)-induced inflammatory bowel disease (IBD) (Hu et al., 2020). A recent study revealed that the presence of *Ruminococcaceae UCG-002* in the intestine affects the structure and function of gut microbiota, which leads to IgE-mediated food allergies (Lee et al., 2021). The results of the present study revealed that the abundance of *V*. sp. in the intestine of patients with colon cancer, who were not infected with *P. hominis*, was significantly reduced in comparison to that of healthy individuals without colon cancer. *V*. sp. is a gram-negative anaerobic micrococcus that thrives in the oral cavity, respiratory tract, and intestine of humans and animals. Adenomatous polyps are the most common precursors of CRC. Studies have demonstrated that the abundance of *V*. sp. can be used to predict the state of adenomas, however, their abundance in the intestine of individuals without adenomas may play a protective role in gut health (Hale et al., 2017). Studies have also reported that the treatment of CRC with XELOX (oxaliplatin + capecitabine) chemotherapy significantly increases the abundance of *V*. sp. in the intestine (Han et al., 2020). These findings confirm that changes in the abundance of *A*. sp., *Le*. sp., *Pa*. sp., *Ruminococcaceae UCG-002*, and *V*. sp. in the intestine are associated with the occurrence of colon cancer.

The characteristics of gut microbiota in patients with colon cancer infected with *P. hominis* and uninfected patients with colon cancer were determined and compared in the present study. The results demonstrated that the relative abundance of the *Eu*. *eligens* group and *Ruminococcaceae UCG-002* was reduced, while the relative abundance of *Fl*. sp., *La*. sp., and *R*. *gnavus* increased in the guts of uninfected patients with colon cancer, compared to that of patients with colon cancer who were infected with *P. hominis*. Previous studies have demonstrated that intestinal worm infections increase the abundance of the *Eu*. *eligens group* in the intestine (Sokolova et al., 2021). In addition, *Eu*. *eligens* is negatively associated with intestinal inflammation (Shao and Zhu, 2020). *Fl.* sp. can metabolize SCFAs. Previous studies have reported that the transplantation of feces from people who ingested rice bran into mice with azomethine/DSS (AOM/DSS)-induced CRC significantly inhibited the development of CRC, and the abundance of *Fl*. sp. and *Oscillibacter* sp. in murine intestine increased significantly following transplantation (Parker et al., 2021). Berberine inhibits AOM/DSS-induced CRC in mice by regulating gut microbiota and this protective effect is primarily associated with the increased abundance of SCFA-producing bacteria such as *Fl*. sp. and *O*. sp. (Chen et al., 2020). However, some reports have also demonstrated that *Fl*. sp. may serve as a diagnostic marker for Crohn’s disease (Mancabelli et al., 2017). *La*. sp. can synthesize butyric acid through the 4-aminobutyric acid/succinic acid pathway (Vital et al., 2014). Previous studies have reported that the abundance of *O*. sp. and *La*. sp. significantly increases in the intestine of mice with AOM/DSS-induced colon cancer and *Saccharomyces boulardii* inhibits colon cancer by inhibiting the relative abundance of *La*. sp. and *O*. sp. in the gut (Wang et al., 2019). Two surveys on 1,012 volunteers (including 274 patients with CRC, 353 patients with adenomas, and 385 control individuals) and 676 volunteers (including 210 patients with CRC, 115 patients with advanced adenoma, 86 patients with non-advanced adenoma, and 265 control individuals) separately demonstrated that the presence of *La*. sp. (m3) in the feces can be used for diagnosing adenoma and CRC (Liang et al., 2020; Liang et al., 2021). *R*. *gnavus* is a gram-positive bacterium that is usually considered to be pathogenic in the intestinal tract. Studies have reported that the abundance of *R*. *gnavus* is significantly higher in the intestines of children with type I diabetes and IBD (Hall et al., 2017; Abdellatif et al., 2019). These findings indicate that infection with *P. hominis* may increase susceptibility to colon cancer by inhibiting the abundance of gut probiotics, including the *Eu*. *eligens* group, and promoting the abundance of gut pathogenic bacteria associated with colon cancer, including *La*. sp. and *R*. *gnavus*.

It has been reported that the host immunity caused by parasitic infections are related to the regulation of host intestinal flora (Dupaul-Chicoine et al., 2013). The regulation of intestinal microflora by the *P. hominis* infection may be similar to the intestinal microflora imbalance caused by infection with other parasitic protozoa (such as *Giardia duodenum*, *Cryptosporidium parvum*, and *Blastocystis* sp.),. For example, *Giardia duodenum* infection leads to persistent disruption of intestinal flora, which activates the TLR4 signaling pathway and promotes the production of inflammatory cytokine IL-1β (Beatty et al., 2017). There are several changes occurring in the gut microbiota structure when *Cryptosporidium parvum* induces Th1-driven MyD88 immune response, resulting in the promotion of the inflammatory response to produce pro-inflammatory cytokines IFN-γ and IL-12 (Chappell et al., 2016). Infection with *B. hominis* not only elicits a strong host immune response, but also alters the structure and diversity of gut microbiota in healthy humans (Audebert et al., 2016). However, the regulatory mechanism underlying the association among gut microbiota, *P. hominis*, and colon cancer requires further elucidation. In addition, further in-depth studies are necessary to determine the complex issue of whether patients with colon cancer are first infected with *P. hominis*, which subsequently induces colon cancer, or whether patients with colon cancer are prone to infection with *P. hominis*, causing the weakening of immune function. The results of this study indicated that intestinal infection with *P. hominis* may aggravate the development of colon cancer. It is therefore crucial to improve the clinical detection of *P. hominis* infections for reducing the incidents of colon cancer in future.

In conclusion, the results of this study demonstrated that *P. hominis* infections reduced the abundance of gut probiotics, including the *Eu*. *eligens* group, while the increased incidence of colon cancer was associated with a higher abundance of pathogenic bacteria such as *La*. sp. and *R*. *gnavus*. The results suggested that infection with *P. hominis* can aggravate the development of colon cancer. It is therefore necessary to place emphasis on the detection of *P. hominis* in clinical practice. The results of this study can serve as important references in determining the pathogenesis, diagnosis, and development of precision medicine for colon cancer.

## Data availability statement

The datasets presented in this study can be found in online repositories. The names of the repository/repositories and accession number(s) can be found below: The data that support the findings of the study is openly available in Sequence Read Archive (SRA) at NCBI with reference number PRJNA781769.

## Ethics statement

The studies involving human participants were reviewed and approved by the Ethics Committees of Jilin Cancer Hospital and the Second Hospital of Jilin University (Reference No. 201712-047). Written informed consent for participation was not required for this study in accordance with the national legislation and the institutional requirements.

## Author contributions

HZ and YY carried out the experiments and investigation, XY and YC provided the resources. HZ, XW, XL, and PG wrote the original draft. JL and NZ wrote the review and edited the paper. XZ and NZ conceived the experiment. All authors contributed to the article and approved the submitted version.

## Funding

This research was funded by the National Natural Science Foundation of China (No.32102696), and the National Key Research and Development Program of China (No.2021YFF0702900).

## Acknowledgments

The authors would like to acknowledge Dr. Xiaoyu Hu from College of Veterinary Medicine, Jilin University, for the assistance with bioinformatics analysis.

## Conflict of interest

The authors declare that the research was conducted in the absence of any commercial or financial relationships that could be construed as a potential conflict of interest.

## Publisher’s note

All claims expressed in this article are solely those of the authors and do not necessarily represent those of their affiliated organizations, or those of the publisher, the editors and the reviewers. Any product that may be evaluated in this article, or claim that may be made by its manufacturer, is not guaranteed or endorsed by the publisher.
