# Supplementary material for: Changes of gut microbiota in colorectal cancer patients with Pentatrichomonas hominis infection
Source: Front Cell Infect Microbiol. 2022 Aug 31;12:961974. doi: 10.3389/fcimb.2022.961974 (PMC9471007; doi:10.3389/fcimb.2022.961974)
Supplement: Supplementary file 1 [file Image_1.pdf]

Supplementary Material

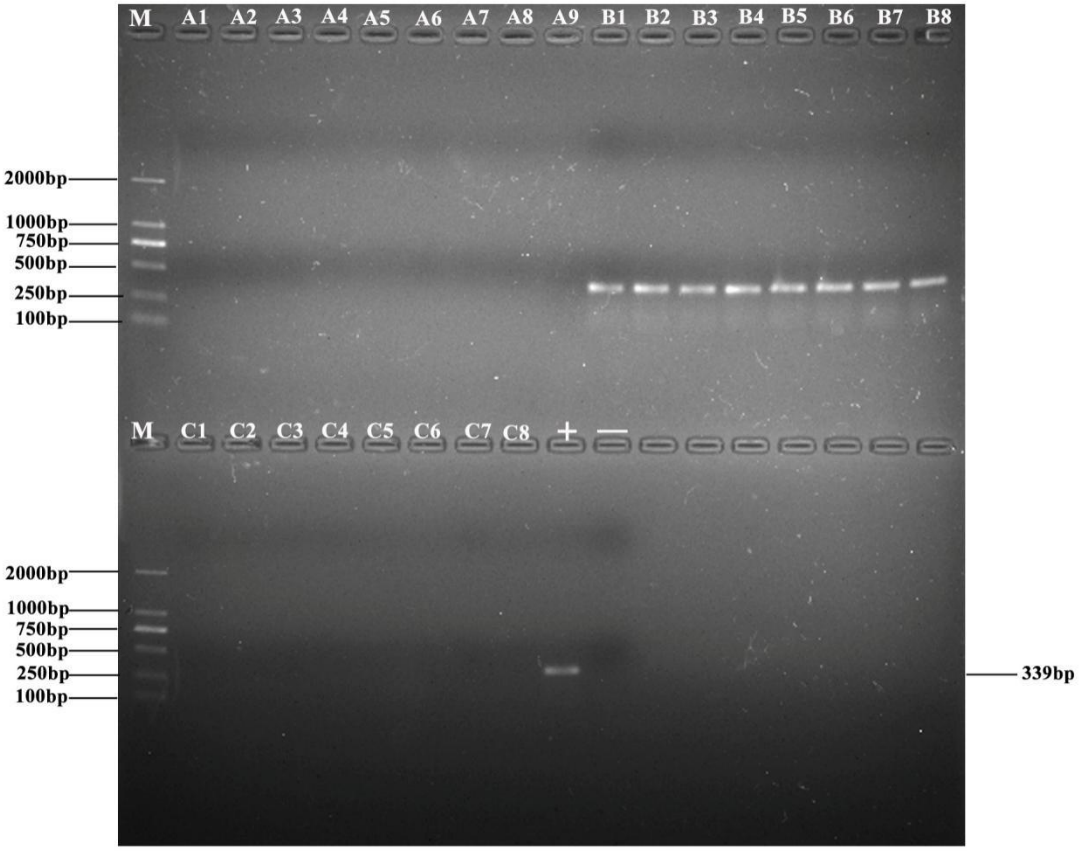

Fig. S1 The PCR electropherogram for detection of the partial 18S rRNA gene of *P. hominis* in fecal samples. 25 fecal samples (9 controls, 8 colon cancer infected with *P. hominis* patients, and 8 colon cancer without infected with *P. hominis* patients) were examined by nested PCR using the partial 18S rRNA gene as target gene (Li et al. 2016).

M represented DL2000 DNA Marker. Lanes+ represented positive control, Lanes- represented negative control.Lanes A1-A9 indicated 9 controls sample detected.Lanes B1-A8 indicated 8 colon cancer infected with *P. hominis* patients sample detected.Lanes C1-C8 indicated 8 colon cancer without infected with *P. hominis* patients sample detected.

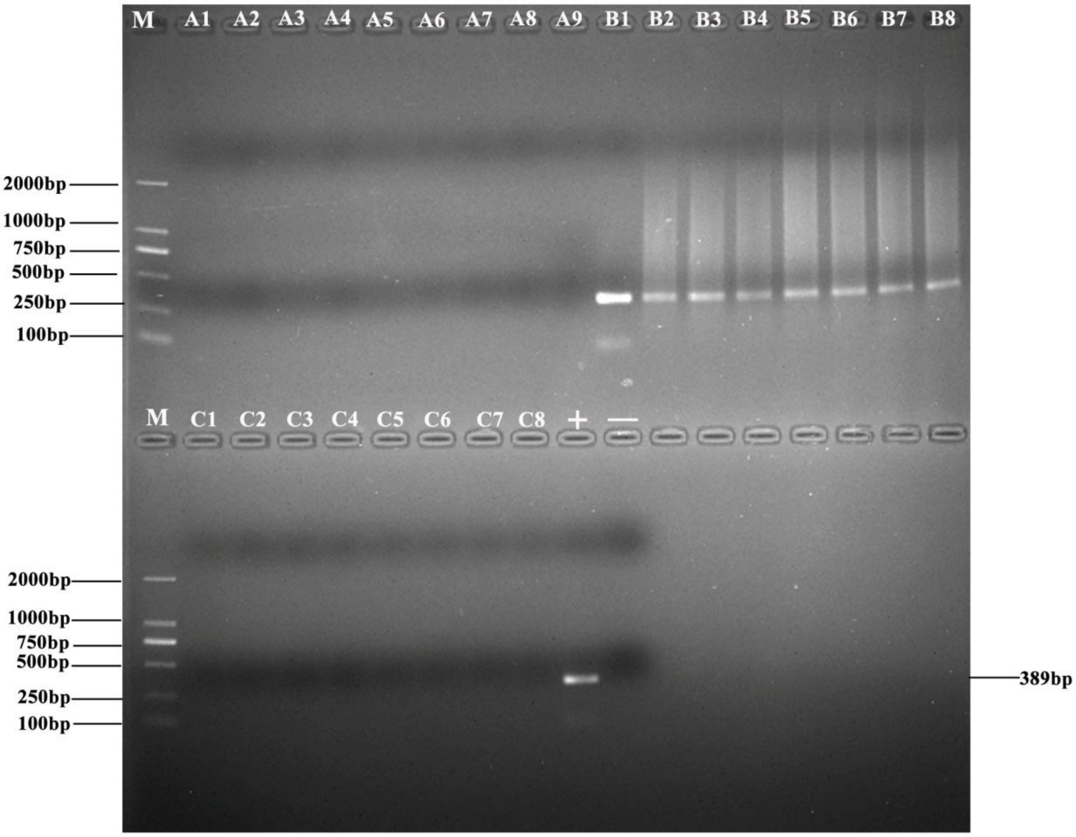

Fig. S2 The PCR electropherogram for detection of the partial ITS sequences of *P. hominis* in fecal samples. 25 fecal samples (9 controls, 8 colon cancer infected with *P. hominis* patients, and 8 colon cancer without infected with *P. hominis* patients) were examined by nested PCR using the partial ITS sequences as target gene (Kamaruddin et al. 2014).

M represented DL2000 DNA Marker. Lanes+ represented positive control, Lanes- represented negative control.Lanes A1-A9 indicated 9 controls sample detected.Lanes B1-A8 indicated 8 colon cancer infected with *P. hominis* patients sample detected.Lanes C1-C8 indicated 8 colon cancer without infected with *P. hominis* patients sample detected.

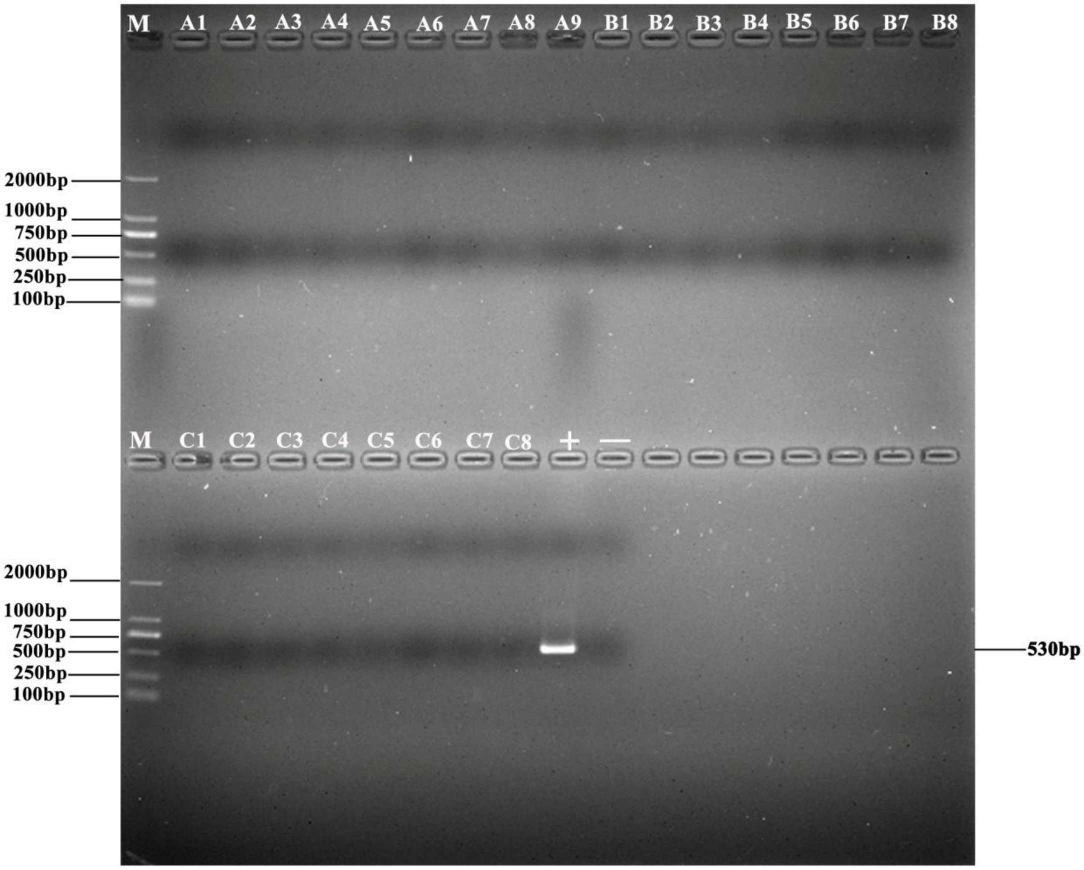

Fig. S3 The PCR electropherogram for detection of the TPI gene of *G. duodenalis* in fecal samples. 25 fecal samples (9 controls, 8 colon cancer infected with *P. hominis* patients, and 8 colon cancer without infected with *P. hominis* patients) were examined by nested PCR using the TPI gene as target gene (Sulaiman *et al*. 2003).

M represented DL2000 DNA Marker. Lanes+ represented positive control, Lanes- represented negative control. Lanes A1-A9 indicated 9 controls sample detected.Lanes B1-A8 indicated 8 colon cancer infected with *P. hominis* patients sample detected.Lanes C1-C8 indicated 8 colon cancer without infected with *P. hominis* patients sample detected.

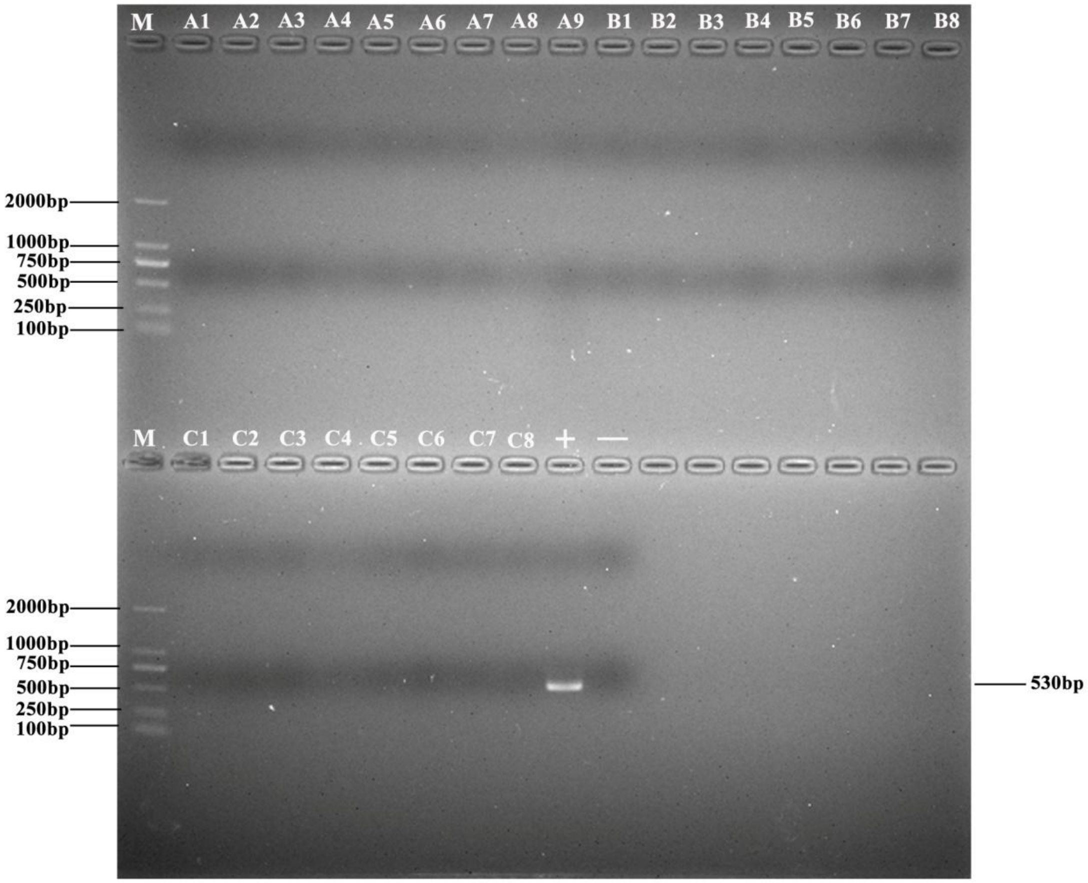

Fig. S4 The PCR electropherogram for detection of the GDH gene of *G. duodenalis* in fecal samples. 25 fecal samples (9 controls, 8 colon cancer infected with *P. hominis* patients, and 8 colon cancer without infected with *P. hominis* patients) were examined by nested PCR using the GDH gene as target gene (Caccio` et al., 2008).

M represented DL2000 DNA Marker. Lanes+ represented positive control, Lanes- represented negative control.Lanes A1-A9 indicated 9 controls sample detected.Lanes B1-A8 indicated 8 colon cancer infected with *P. hominis* patients sample detected.Lanes C1-C8 indicated 8 colon cancer without infected with *P. hominis* patients sample detected.

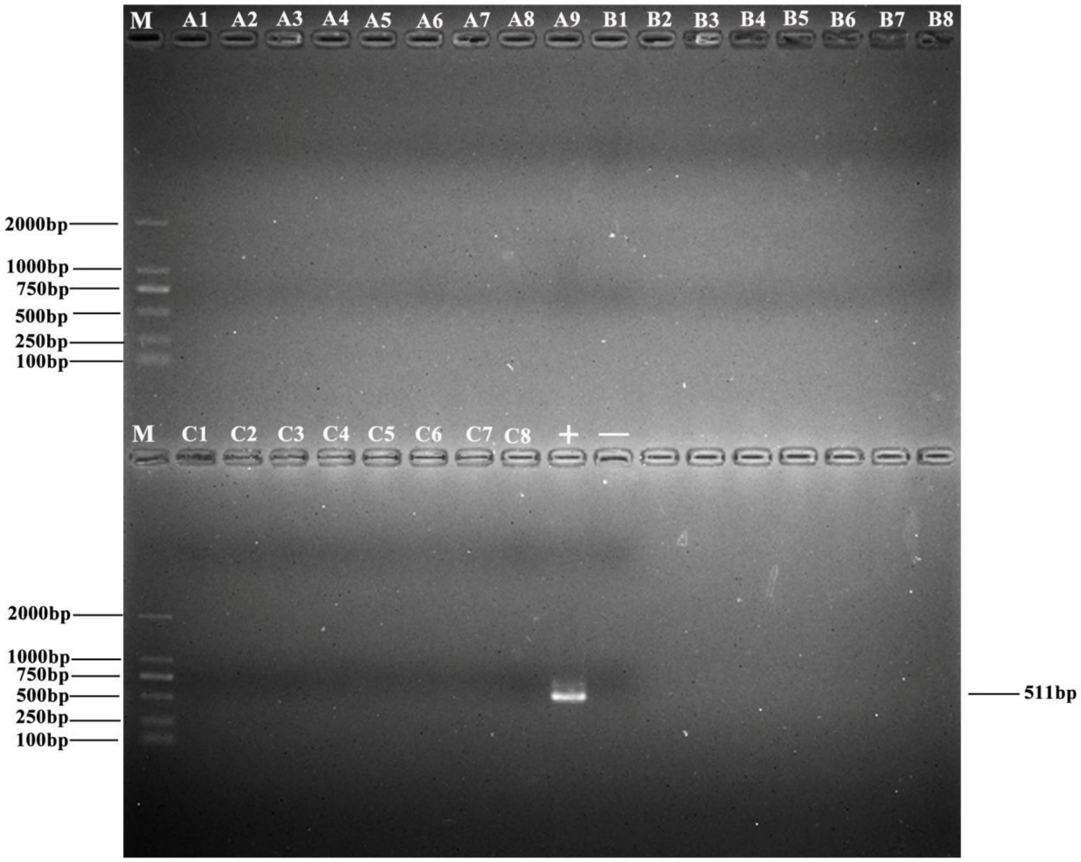

Fig. S5 The PCR electropherogram for detection of the β-giardin gene of *G. duodenalis* in fecal samples. 25 fecal samples (9 controls, 8 colon cancer infected with *P. hominis* patients, and 8 colon cancer without infected with *P. hominis* patients) were examined by nested PCR using the β-giardin gene as target gene (Lalle et al., 2005).

M represented DL2000 DNA Marker. Lanes+ represented positive control, Lanes- represented negative control. Lanes A1-A9 indicated 9 controls sample detected. Lanes B1-A8 indicated 8 colon cancer infected with *P. hominis* patients sample detected. Lanes C1-C8 indicated 8 colon cancer without infected with *P. hominis* patients sample detected.

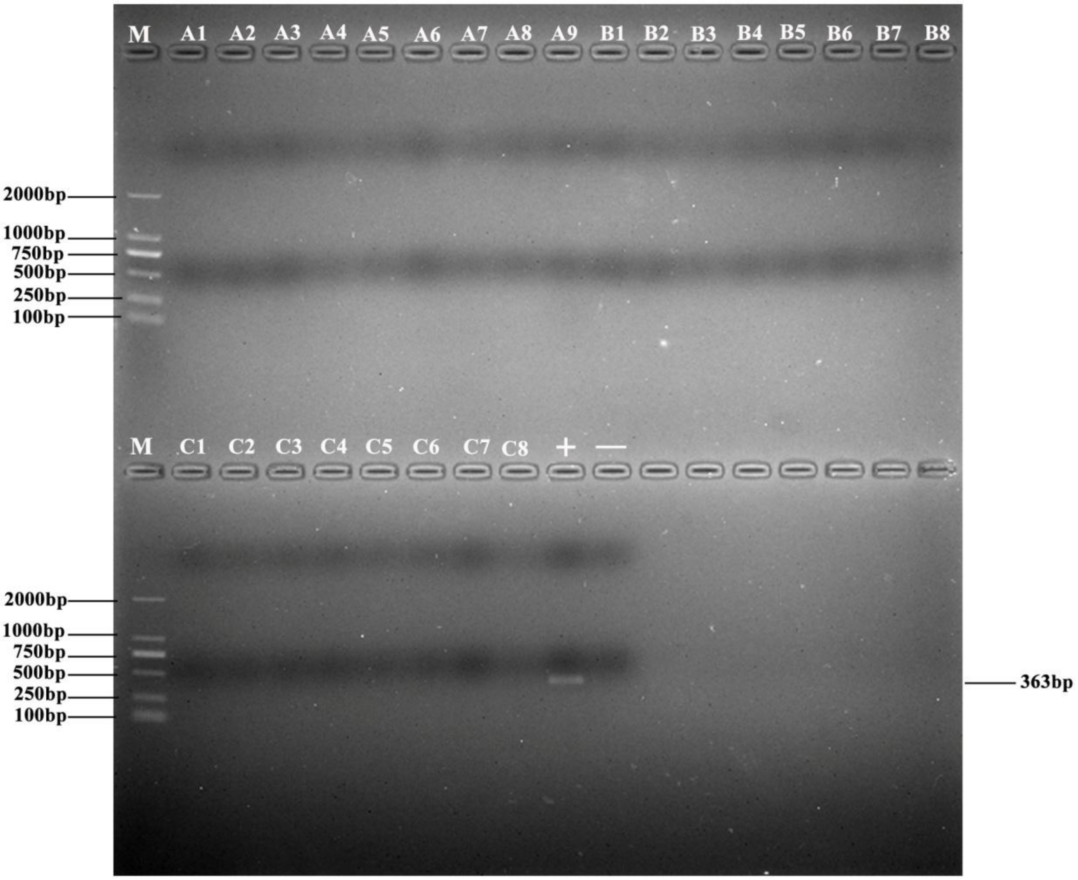

Fig. S6 The PCR electropherogram for detection of the gp60 sequences of *C. parvum* in fecal samples. 25 fecal samples (9 controls, 8 colon cancer infected with *P. hominis* patients, and 8 colon cancer without infected with *P. hominis* patients) were examined by nested PCR using the gp60 sequences as target gene (Hijjawi et al., 2017).

M represented DL2000 DNA Marker. Lanes+ represented positive control, Lanes- represented negative control. Lanes A1-A9 indicated 9 controls sample detected. Lanes B1-A8 indicated 8 colon cancer infected with *P. hominis* patients sample detected.Lanes C1-C8 indicated 8 colon cancer without infected with *P. hominis* patients sample detected.

The following original literatures are the detection of *P. hominis*, *G. duodenalis* and *C. parvum* in fecal samples for the nested PCR.

References

Cacciò, S.M., Beck, R., Lalle, M., Marinculic, A., Pozio, E. (2008). Multilocus genotyping of *Giardia duodenalis* reveals striking differences between assemblages A and B. International Journal for Parasitology. 38(13):1523-1531. doi:10.1016/j.ijpara.2008.04.008

Hijjawi, N., Zahedi, A., Kazaleh, M., Ryan, U.(2017). Prevalence of *Cryptosporidium* species and subtypes in paediatric oncology and non-oncology patients with diarrhoea in Jordan. Infection Genetics & Evolution. 55:127-130. doi:10.1016/j.meegid.2017.08.033

Kamaruddin, M., Tokoro, M., Rahman, M.M., Arayama, S., Hidayati, A.P.N., Syafruddin, D., et al. (2014). Molecular characterization of various trichomonad species isolated from humans and related mammals in Indonesia. Korean Journal of Parasitology. 52(5): 471-478. doi: 10.3347/kjp.2014.52.5.471

Lalle, M., Pozio,E., Capelli, G., Bruschi, F., Crotti, D., Cacciò, S.M.(2005). Genetic heterogeneity at the β-giardin locus among human and animal isolates of Giardia duodenalis and identification of potentially zoonotic subgenotypes. International Journal for Parasitology. 35(2):207-213. doi: 10.1016/j.ijpara.2004.10.022

Li, W.C., Ying, M., Gong, P.T., Li, J.H., Yang, J., Li, H., et al. (2016). *Pentatrichomonas hominis*: prevalence and molecular characterization in humans, dogs, and monkeys in Northern China. Parasitology Research. 115(2):569-574. doi: 10.1007/s00436-015-4773-8

Sulaiman, I.M., Fayer,R., Bern, C., Gilman, R.H., Trout, J.M., Schantz, P.M.,et al. (2003).Triosephosphate isomerase gene characterization and potential zoonotic transmission of Giardia duodenalis. *Emerging Infectious Diseases*. 9(11):1444-52. doi: 10.3201/eid0911.030084.
